# Comparison of different proxy approaches to determine the need for specialized palliative care in patients with incurable cancer

**DOI:** 10.1186/s12904-026-02106-z

**Published:** 2026-05-06

**Authors:** Nikola Reichel, Maria Heckel, Susanne Gahr, Christoph Ostgathe

**Affiliations:** 1 Department of Palliative Medicine, Universitätsklinikum Erlangen, Friedrich-Alexander-Universität Erlangen-Nürnberg (FAU), Erlangen, Germany; 2 CCC Erlangen-EMN: Comprehensive Cancer Center Erlangen-EMN (CCC ER-EMN), Erlangen, Germany

**Keywords:** Palliative care, Cancer, SPC need, Surprise question, ECOG, NCCN screening tool

## Abstract

**Background:**

Patients suffering from cancer can benefit from a timely integration of palliative and end-of-life care. In the literature different approaches are discussed that can be used by health care professionals (as proxies) to determine cancer patients in need for specialist palliative care. Until now data on comparing different tools is scarce. This study compared published methods for detecting patients with advanced and incurable cancer in need for specialist palliative care.

**Methods:**

Data of three hundred and sixteen patients with incurable cancer—collected during a study validating the German version of a screening tool based on NCCN guidelines (Glare) — were used for secondary analysis. The data were used to test the performance of different tools in detecting patients with palliative care needs: two disease-specific classifications (Gaertner, Benthien), the Eastern Cooperative Oncology Group Performance Status (ECOG), the Surprise Question, as well as a combination of the Surprise Question and the German NCCN tool and the Surprise Question and the ECOG score. To quantify which tool performed best, survival, Integrated Palliative Outcome Scale (IPOS – staff version) (one or more items ≥ 3), and the information of a preexistent contact to palliative care served as indicators of real SPC needs in this patient group.

**Results:**

The combination of Surprise Question and the German NCCN Screening tool showed a sensitivity between 71.5%–94.3% and specificity between 56.0%–91.3%, while the combination of Surprise Question and ECOG score had a sensitivity between 37.4%–75.7% and specificity between 86.2%–100%. Benthien’s classification performed a fair sensitivity (74.8%–91.5%) and a weak specificity (27.3%–39.4%), whereas the guidelines by Gaertner showed high sensitivity (92.2%–100%), but very low specificity in all standards (0.0%–9.9%).

**Conclusion:**

While the combination of the Surprise Question and the German NCCN screening tool showed the best results in terms of sensitivity and specificity overall, a combination of the Surprise Question and ECOG score proved to be highly specific and as time-efficient in identifying patients in need of SPC, which may be beneficial.

**Supplementary Information:**

The online version contains supplementary material available at 10.1186/s12904-026-02106-z.

## Background

Patients suffering from cancer can benefit from a timely integration of specialized palliative care (SPC). Previous studies have shown that quality of life, mood, and symptom intensity of patients are better with early integration of palliative care compared to standard care [1–3]. Survival time when palliative care is integrated early in the disease trajectory is at least unchanged [3], some studies even suggest an advantage of survival [1, 4]. Despite these advantages and different recommendations regarding the timing of integration of palliative care, there is no established screening tool or disease-specific approach to detect the patients with cancer in need of SPC. The gold standard would be a self-assessment, hence in palliative care due to cognitive impairment and/or reduced physical abilities many patients are not able to use patient reported outcomes (PROM). Therefore, also proxy measures are widely discussed. This study compared different approaches that can be used by health care professionals (as proxies) to determine cancer patients in need for specialist palliative care: a screening procedure [5], which is based on a tool developed by Glare and Chow using the guidelines of the National Comprehensive Cancer Network (NCCN) [6], disease-specific guidelines by Gaertner [7], a disease-specific classification by Benthien [8], the ECOG score [9], the Surprise Question [10], a combination of the Surprise Question and the German NCCN screening tool [5], and a combination of the Surprise Question and the ECOG score.

## Methods

### Description of the different approaches

We compared the following approaches as predictor variables to estimate the need for specialized palliative care (SPC):

#### German NCCN screening tool

The German NCCN screening tool is a patient-centered screening instrument containing ten items (diagnosis, functional status (ECOG score), complications of the disease, comorbidities, uncontrolled symptoms, distress in patient or family, their concerns about course of disease and decision making, their request of palliative care consultation, support of medical team, prolonged length of stay); its score ranges from 0 to 14 points. In the validation study, before screening, patients were filtered by the criteria “advanced and incurable cancer” and the Surprise Question (“I would be surprised if the patient died within 1 year”). Only if a patient’s disease was advanced and the Surprise Question was answered “no”, the full NCCN screening was applied via proxy assessment by physicians. A cut-off score of ≥ 5 to define the SPC need was determined [5].

#### Benthien

Benthien’s approach developed for the DOMUS study mainly used lines of therapy (e.g., “Refractory to 2nd line treatment of metastatic or advanced disease”) to define “limited antineoplastic treatment options” for nine different tumor entities (e.g., head and neck cancer, breast cancer, gastrointestinal cancer) to classify the palliative care population [8].

#### Gaertner

Gaertner developed guidelines for 19 different cancer types to give disease-specific definitions of when palliative care should be initiated. The stage of a disease (e.g., metastasized and inoperable for breast cancer, locally progressive and inoperable for cervical cancer), or in some cases therapy options (e.g., if intravenous chemotherapy or bone marrow transplantation is not indicated anymore for AML) provide specific recommendations for the early integration of palliative care [7].

#### Surprise question

The Surprise Question (“Would I be surprised if this patient dies within 12 months?”) is a screening tool to identify patients who may need palliative care. If it is answered “no”, the patient is screened positive [10].

#### ECOG score

An ECOG score of 3–4 points was determined as “SPC need”, while 0–2 points were seen as “no SPC need”.

#### German NCCN screening tool plus surprise question

The combination between the German NCCN screening tool and the Surprise Question was interpreted as follows: If the Surprise Question was answered “yes”, the patient was listed as “no SPC need” and a screening with the German NCCN screening tool was not performed. If the Surprise Question was answered “no”, the patient was screened with the NCCN screening tool: A screening score 0–4 was then seen as “no SPC need”; a score 5 or higher was transferred as “SPC need”.

#### ECOG score plus surprise question

The combination of the ECOG score and the Surprise Question was interpreted likewise. A need for SPC was seen when the Surprise Question was answered “no” and the ECOG score was 3 or 4 points, otherwise the patient was classified as “no SPC need”.

### Study design and sampling

The study at hand is a secondary analysis of adult advanced cancer patients’ data sets (*n* = 320) of the original validation study of a screening procedure based on proxy assessment by physicians in university hospital wards (respiratory medicine, hematology/oncology, gynecology and palliative medicine) between January and August of 2017 and patient records. On admission of the patient, health care professionals assessed the patient if the disease was incurable and if the Surprise Question was answered “no”. Only if a patient’s disease was advanced and the Surprise Question was answered “no”, the full NCCN screening was applied via proxy assessment by physicians [5]. This study included all patients assessed by the Surprise Question regardless of how the Surprise Question was answered. Since the Surprise Question was only answered if the patient had advanced oncological disease, this study only included data of patients with advanced and incurable cancer.

**Fig. 1:**
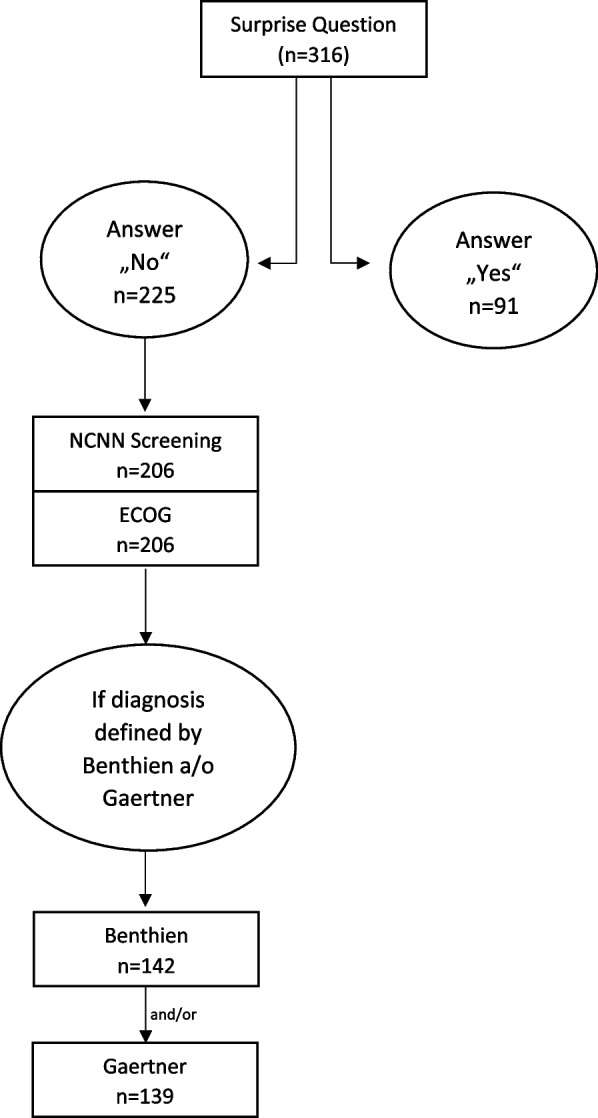
Flowchart of patient inclusion and subgroup allocation

We included all 320 patients assessed by the Surprise Question and followed-up their survival time. The survival time served as an indicator for SPC need. If not available through the screening study records, physicians provided information on whether and when patients had died during the hospital stay. If information on death was not available through search of a public obituary, the local residents’ registration office was contacted in October 2021 to obtain a possible date of death or the patient’s current and registered information. This approach is consistent with German law, which allows residents’ registration offices to provide this information if research is conducted. We censored the patients on October 1, when according to the local residents’ registration office they were still alive. Out of the 320 included patients, 208 were screened by the German NCCN. Data of these 208 patients about the tumor stadium, line of therapy, possible progress and further information (if needed) were extracted from the patient files and were used to apply the approaches by Gaertner and Benthien. According to the principal of data avoidance we only used patient records and personal data when necessary. An overview of the process is provided in the flowchart (Fig. 1).

The figure illustrates the screening and assessment process based on the Surprise Question (*n* = 316). Patients with a “No” response (*n* = 225) underwent further evaluation, including the NCCN screening tool and ECOG performance status (*n* = 206). Subgroups were further classified according to the Benthien (*n* = 142) and Gaertner (*n* = 139) criteria, depending on the underlying diagnosis. Patients with a “Yes” response to the Surprise Question (*n* = 91) were not further assessed using these tools.

### Different standards and SPC needs

Since there is still no gold standard for SPC need, six different indicators of SPC served as a standard to quantify which instrument is most precise to determine SPC need. The first standard was one or more items with 3 or 4 points in the Integrated Palliative Outcome Scale (IPOS–staff version), indicating that the patient had a symptom that needed high clinical attention and therefore indicated SPC need [11]. The IPOS score was assessed only for patients screened by the NCCN screening, which was only performed if the Surprise Question was answered “no”. The second standard was the preexistent contact to palliative care, which was documented for all included patients. In case the patient was already in contact with palliative care—including a palliative care unit or an inpatient and outpatient consultation team—this served also as an indicator that SPC was needed and should be detected by the different methods. The third standard was defined as dying within 30 days, the fourth standard as dying within one year, and respectively indicated SPC need. The fifth standard was a combination of the previous ones to summarize the previous indicators: if a patient had one or more IPOS items with a score of 3 or 4 points, or was already in contact with a palliative care/consultation team, or died within 30 days—if at least one of the criterion applied—it indicated SPC need. The sixth standard worked in the same way and used the combination one or more IPOS items with a score of 3 or 4 points, or was already in contact with a palliative care/consultation team, or died within one year, then it indicated SPC need.

### Statistical consideration

The Kaplan-Meier method and log-rank test were conducted for each tool separately to calculate if there was a significant difference in survival between the patient groups rated “no SPC need” and “SPC need”.

For each method, the chi-square test was performed to check for contingency between SPC need and dying within 30 days, as well as dying within a year. Moreover, the contingency in-between any combination of the different instruments—the German NCCN screening tool, Benthien, Gaertner and the ECOG score—was calculated to see if the different methods pointed in the same direction regarding SPC need. In case of a significant contingency, the Phi-coefficient was calculated to measure the association between two variables. The Phi-coefficient (φ) is used to quantify the strength of association between two binary variables. It ranges from –1 (perfect negative association) to + 1 (perfect positive association), with 0 indicating no association. In this study, only positive associations are relevant, so values typically range from 0 to 1. Although not equivalent, its range can be loosely compared to the AUC in ROC analysis for orientation purposes, where higher values indicate stronger associations.

The Mann-Whitey-U test was chosen to examine if the screening score differed between the groups “SPC need” and “no SPC need” according to Benthien and Gaertner as well as between the group of patients who died within 30 days versus the group of patients who lived longer than 30 days, respectively a year.

All methods were measured against all six standards to calculate sensitivity, specificity, positive predictive value (PPV) and negative predictive value (NPV).

A statistically significant finding was defined as a two-sided value of *p* < 0.05. For all analyses, SPSS version 28 was used.

#### Ethics

Ethical approval of the local ethics committee was obtained (Ethik-Kommission der Friedrich-Alexander-Universität Erlangen-Nürnberg, 13_17 Bc, 10.02.2017), and cooperating wards had given written consent to this study. The ethics committee did not consider it necessary to obtain declaration of consent of patients to participate, because the project did not include any changes in care or treatment of patients receiving cancer care. Based on the cooperation agreements with the participating wards the follow-up was covered by the state law (Bavarian Hospital Act, §27), according to which hospital doctors may use patient data insofar as this is necessary for research purposes in the hospital.

## Results

### Study population and prevalence

The characteristics of each patient group together with the prevalence of SPC need in each method are described in Table 1: Patient Characteristics of study population and prevalence of SPC need for each investigated method.

**Table 1:** Patient Characteristics of study population and prevalence of SPC need for each investigated method

Method	Number of patients	Sex	Mean age in years (standard deviation)	Prevalence of SPC need
German NCCN	206	54.4% female 45.6% male	63.6 (13.3)	80.6%
Benthien	142	62.0% female 38.0% male	63.0 (12.1)	74.6%
Gaertner	139	64.7% female 35.3% male	62.1 (13.4)	92.8%
Surprise Question/Overall Population	316	60.4% female 39.6% male	63.0 (13.4)	71.2%
ECOG score	206	54.4% female 45.6% male	63.6 (13.3)	40.8%
German NCCN plus Surprise Question	297	59.9% female 40.1% male	62.8 (13.4)	55.9%
ECOG plus Surprise Question	297	59.9% female 40.1% male	62.8 (13.4)	27.9%

Three Hundred Twenty patients with advanced and incurable cancer were identified. 60.6% were female and 39.4% were male. The age range spanned from 19 to 96 years with a mean age of 63.0 years. Four patients were excluded due to missing traceability. Sixteen patients were included twice, because they were admitted to the hospital at an interval of more than three months.

The date of death of 77 patients was documented within the original validation study data, 56 in the patient records. In 82 cases, a public obituary was found, in which full name and date of birth matched the patient’s information. In 95 cases, the local registration office could give a date of death or provide the information that the patient was currently registered, and we listed them as alive. In three cases, the local registration office only had the date of the patient’s emigration into another country, thus they were censored at this date.

Diagnoses included and excluded according to the recommendations of Benthien and Gaertner are listed in Table 6 – Table 9 in the Supplement. The medical files of seven patients regarding Benthien and one regarding Gaertner showed a general deterioration of the patient’s condition and the progress of cancer retrospectively seemed likely but was not documented. In these cases, the progress would qualify the patient for “SPC need”, no progress would equal “no SPC need”. Since a distinct decision was retrospectively not possible, we excluded these patients from further analysis. Three cases according to the recommendations of Benthien and one case regarding the recommendations of Gaertner had to be excluded due to incomplete or missing information in the patients’ records.

For 19 patients, the Surprise Question was answered “no”, but the German NCCN screening tool was either not performed, not completed, or not performed in time and the data set was excluded. Hence, we excluded also them from the analysis of the combined methods “Surprise Question plus Screening score” and “Surprise Question plus ECOG score”.

For 306 patients it was documented, if there was a preexisting contact with palliative care or not; 72 of them already were in contact with palliative care. For 234 patients, no contact was established. In 67.0% of the screened patients, the IPOS score was not filled out completely, but more than half of the data was always completed. If one or more IPOS items were scored 3 or 4 points, it was interpreted as an indicator for SPC, even if items were missing.

### Survival time analysis

Median survival and log-rank tests were performed for each method to determine whether significant differences existed between patients with “SPC need” and “no SPC need”. The results are summarized in Table 2: Results of log-rank tests for each investigated approach. Median survival times reported in Table 2 were estimated using the Kaplan-Meier method, which accounts for censoring. Kaplan-Meier survival curves are included in the supplementary material as Figs. 2–8.

**Table 2:** Results of log-rank tests for each investigated approach

Method	Chi-Quadrat (degrees of freedom)	Value of *p*	Median survival (days)	
German NCCN	10.636 (1)	0.001	No SPC need	237
			SPC need	45
			Overall	66
Benthien	11.476 (1)	<.001	No SPC need	215
			SPC need	48
			Overall	80
Gaertner	1.539 (1)	0.215	No SPC need	218
			SPC need	82
			Overall	84
Surprise Question	67.416 (1)	<.001	No SPC need	915
			SPC need	74
			Overall	141
ECOG score	60.871 (1)	<.001	No SPC need	188
			SPC need	15
			Overall	66
Surprise Question plus German NCCN	81.418 (1)	<.001	No SPC need	548
			SPC need	45
			Overall	141
Surprise Question plus ECOG score	128.357 (1)	<.001	No SPC need	294
			SPC need	15
			Overall	141

### Screening score analysis

While there was no significant difference between the group “SPC need” (mean score: 6.96 points) and “no SPC need” (mean score: 7.20 points) according to Gaertner (U = 630.000; Z = −0.123; *p* = 0.902), a significant difference was found in the different groups (“SPC need” mean score: 7.41 points, “No SPC need” mean score: 5.97 points) according to Benthien (U = 1299.000; Z = −2.874; *p* = 0.004).

The Mann–Whitney-U test also showed a significant difference in the screening score of patients who died within 30 days (mean score: 8.92 points) and patients who lived longer than 30 days (mean score: 6.14 points) (U = 1840.000; Z = −7.510, *p* < 0.001) as well as in the screening score of patients who lived longer than a year (mean score: 5.42 points) and patients who died within (mean score: 7.60 points) (U = 1780.500; Z = −4.919; *p* < 0.001).

### Associations between approaches, different approaches and death

The calculations for contingency using chi-square test and Phi-coefficient in-between methods as well as the contingency between each method and death within 30 days and one year are shown in Table 3: Associations between the different approaches used to identify a need for specialized palliative care.

**Table 3:** Associations between the different approaches used to identify a need for specialized palliative care

		**German NCCN**	**Benthien**	**Gaertner**	**Surprise Question**	**German NCCN + Surprise Question**	**ECOG**	**ECOG + Surprise Question**
Benthien	Number of cases	142	-	-	-	-	142	-
	Chi-sq. (df)	2.573 (1)	-	-	-	-	15.505 (1)	-
	Value of p	.109	-	-	-	-	** <.001**	-
	Phi-coefficient	-	-	-	-	-	.330	-
Gaertner	Number of cases	139	114	-	-	-	139	-
	Chi-sq. (df)	*.770 (1)	*.726	-	-	-	*.076 (1)	-
	Value of p	.380	.394	-	-	-	.783	-
	Phi-coefficient	-	-	-	-	-	-	-

^*^Fisher’s exact test was performed, *df* degree of freedom

 and Table 4: Association between the identified need for palliative care (according to each method) and actual patient mortality within 30 days and within one year.

**Table 4:** Association between the identified need for palliative care (according to each method) and actual patient mortality within 30 days and within one year

		**German NCCN**	**Benthien**	**Gaertner**	**Surprise Question**	**German NCCN + Surprise Question**	**ECOG**	**ECOG + Surprise Question**
Dying w. 30 days	Number of cases	206	142	139	316	297	206	297
	Chi-sq. (df)	17.916 (1)	10.529 (1)	*1.969 (1)	21.901 (1)	44.483 (1)	51.764 (1)	78.799 (1)
	Value of *p*	** <.001**	.001	.161	** <.001**	** <.001**	** <.001**	** <.001**
	Phi-coefficient	.295	.272	-	.263	.387	.501	.515
Dying w. one year	Number of cases	204	140	137	313	294	204	294
	Chi-sq. (df)	8.066 (1)	4.230 (1)	.206 (1)	59.023 (1)	54.338 (1)	25.012 (1)	49.053 (1)
	Value of *p*	.005	.040	.650	** <.001**	** <.001**	** <.001**	** <.001**
	Phi-coefficient	.199	.174	-	.434	.430	.350	.408

^*^ = Fisher’s exact test was performed, *df* degree of freedom

In four cases, the expected cell frequencies were below five, thus Fisher’s exact test was performed.

Table 3 presents the associations between the different approaches used to identify a need for specialized palliative care. The Benthien and Gaertner tools were applied only to patients who had received a “no” answer to the Surprise Question. Therefore, comparisons involving these tools are limited to a subset of the cohort. This restriction is reflected in Table 3, where missing values ("–") indicate comparisons that could not be performed on the full sample.

Table 4 displays the association between the identified need for palliative care (according to each method) and actual patient mortality within 30 days and within one year.

### Sensitivity, specificity, PPV and NPV

Sensitivity, specificity, PPV and NPV were calculated for each method with each possible standard. The results are shown in Table 5: Sensitivity, Specificity, PPV, and NPV of each investigated method and standard. Examples of contingency tables used to calculate sensitivity and specificity are provided in the Supplement (Table 10 – Table 15).

**Table 5:** Sensitivity, Specificity, PPV, and NPV of each investigated method and standard

		**German NCCN**	**Benthien**	**Gaertner**	**ECOG**	**German NCCN** ** + Surprise Question**	**Surprise** **Question**	**ECOG** ** + Surprise Question**
IPOS	Sensitivity	87.0	75.2	**93.2**	46.9	-	-	-
	Specificity	58.6	28.6	9.1	96.6	-	-	-
	PPV	92.8	85.8	84.5	98.8	-	-	-
	NPV	42.5	17.7	20.0	23.0	-	-	-
Contact PC	Sensitivity	100	91.3	100	80.3	94.3	94.4	75.7
	Specificity	28.9	33.0	9.9	77.0	56.0	35.5	86.2
	PPV	40.7	40.0	35.4	63.1	40.7	31.1	63.9
	NPV	100	88.6	100	88.9	96.8	95.4	91.7
Dying within 30 days	Sensitivity	96.0	91.5	97.6	73.3	86.7	90.7	65.1
	Specificity	28.2	33.7	9.2	77.9	56.1	36.1	86.4
	PPV	43.4	40.6	31.0	65.5	43.4	34.7	65.1
	NPV	92.5	88.9	90.0	83.6	91.6	91.2	86.4
Dying within one year	Sensitivity	84.5	78.5	93.3	49.1	71.5	85.7	41.1
	Specificity	34.9	39.4	9.1	93.0	73.1	55.5	97.1
	PPV	82.9	80.8	76.4	96.3	82.9	78.0	96.3
	NPV	37.5	36.1	30.0	32.8	58.5	67.8	47.4
IPOS + contact PC + dying within 30 days	Sensitivity	86.6	75.2	93.2	46.9	82.0	94.9	43.9
	Specificity	59.3	28.5	9.1	100	89.8	66.9	100
	PPV	93.4	85.9	85.6	100	93.4	82.2	100
	NPV	40.0	16.7	20.0	22.1	74.0	89.0	50.5
IPOS + contact PC + dying within one year	Sensitivity	83.4	74.8	92.2	43.5	72.5	87.7	37.4
	Specificity	61.5	27.3	0.0	100	93.2	76.3	100
	PPV	97.0	92.5	91.5	100	97.0	91.6	100
	NPV	20.0	8.3	0.0	10.7	53.1	67.8	34.7

## Discussion

To the best of our knowledge, this is the first study to compare different approaches that can be used by health care professionals (as proxies) to determine cancer patients in need for specialist palliative care. The German NCCN screening tool [5], based on Glare and Chow [6], showed significant differences in the survival time between patients with and without SPC needs, the screening score of patients who died within 30 days compared to those who lived longer than 30 days, and the screening score of patients who lived longer than a year or died within. Together with a significant Phi-coefficient showing contingency between SPC-need and dying within 30 days (Phi = 0.295) and one year (Phi = 0.199), and good sensitivity in all standards (83.3%−100%), one can argue that the screening tool is an effective instrument to detect SPC needs with acceptable though not excellent specificity (28.2%−61.5%). The screening tool is the translation and adaptation of a screening tool developed by Glare and Chow [6]. Out of 194 hospitalized patients in MSKCC, New York, GI Oncology Service a total of 34% were screened positive for SPC needs with a cut-off point of 5. Le et al. [12] used the same screening tool translated into Vietnamese to screen hospitalized patients in the Department of Oncology and Palliative Care in Hanoi Medical University Hospital. 44% were screened positive for SPC needs with a score of 5 or more points. Since Le [12] and Glare and Chow [6] didn’t use filter questions like in the German version, the prevalence of SPC need was much lower than in our preselected cohort of patients with negative Surprise Question and advanced incurable cancer (80.8%). If all prescreened patients were included (*n* = 455) the prevalence is at 36.9% and is comparable to the cohort in Hanoi and New York.

Benthien’s [8] classification also showed significant contingency between SPC need and dying within 30 days (Phi = 0.272) and one year (Phi = 0.174), though it was slightly weaker than German NCCN screening tool. Sensitivity and specificity do not perform as well as the screening tool, especially in the combined standards. They reflect fair sensitivity (74.8%−91.5%), but the specificity is weak (27.3%−39.4%). If a patient-based approach is not possible to perform, Benthien’s classification might be an alternative where the decision can be based only on the patient’s medical record. Benthien originally used this approach to classify the palliative care population in a comprehensive cancer center in Copenhagen University Hospital. The prevalence was about 14% and much lower than in our cohort (74.6%). This discrepancy can partially be explained by the preselected patient group we used, which only included patients with a negative Surprise Question and incurable, advanced cancer.

The guidelines proposed by Gaertner [7] showed no significant results, neither in comparing the survival time of the patient groups “SPC need” and “no SPC need” nor in the chi-square test and Fisher’s exact test to find contingency. Together with the high prevalence of SPC need (92.8%) and very low specificity in all standards (0.0%–9.9%), there seems to be no advantage of using this approach in a preselected group of advanced cancer patients. There seems to be no difference if a physician characterizing a patient suffering from “incurable advanced cancer” or is using the guidelines by Gaertner (prevalence of SPC needs 92.8%). If in doubt, the guidelines might help to identify, which patients could be seen as advanced and incurable. But they seem to be too vague to use as a single indicator for SPC needs.

Combining the German NCCN screening tool with the Surprise Question—as done in the original validation study [5]—increases the specificity substantially to a range of 56.1%−93.2%. Additionally, the combination of the Surprise Question and the screening tool shows higher specificity than the Surprise Question alone (35.5%–76.3%), while also maintaining a high level of sensitivity (71.5%−94.9%). Contingency between dying within one year and the Surprise Question (Phi = 0.434), as well as the screening tool plus the Surprise Question (Phi = 0.430) is the strongest compared to the other tools. After all, the combination of the Surprise Question and the screening tool has a high sensitivity (71.5%−94.3%), high specificity (56.0%−93.2%), and seems to be a reasonable precise approach to differentiate between patients with and without SPC needs. Our results show a good sensitivity of the Surprise Question indicating that it could be used as a screening tool for SPC needs in patients with advanced cancer. Although PPV for dying within one year was low (55.5%), the combined standard of dying within one year, IPOS item of 3–4 points and preexisting contact to a PC unit showed high PPV (91.6%). It seems that the Surprise Question not only serves as a good indicator for near death (90.7% sensitivity for dying within 30 days, 85.7% sensitivity withing one year), but also for SPC needs in our cohort of patients with incurable advanced cancer.

Our results are in contrast with the meta-analysis of Downar et al. [13], which states that the Surprise Question is a poor to modest tool to predict death in the next 6–18 months in serious ill patients. In patients with cancer, sensitivity (66.4%) and PPV (46.8%) were low, while specificity (84.3%) and NPV (92.4%) were higher [13].

Davis et al. validated the Surprise Question in cancer patients undergoing a systemic therapy. PPV (54.9%), NPV (67.2%), sensitivity (59.0%), and specificity (62.5%) were low, which led to the conclusion, that the Surprise Question poorly predicts survival [14].

Stoppelenburg et al. found, that the Surprise Question predicts the death in patients with cancer reasonably well. In a prospective study for inpatients with cancer, sensitivity of the Surprise Question predicting death (79%) and PPV (71%) were lower than our results, while specificity (66%) and NPV (75%) were higher [15].

This could lead to the conclusion that the Surprise Question works well as a screening tool for near death and SPC needs, if used with patients with advanced incurable cancer.

While the ECOG score shows low sensitivity (43.5%−80.3%), its specificity is high in all standards (77.0%−100%) and there is a strong contingency between an ECOG score of 3–4 points and dying within 30 days (Phi = 0.501). A combination between the Surprise Question and the ECOG score improves specificity (86.2%−100%), contingency of dying within 30 days (Phi = 0.515), and the contingency of dying within one year (Phi = 0.408). Hence, patients who screened positive with the Surprise Question and have an ECOG score of 3 or 4 points are very likely to have SPC needs. The sensitivity, specificity, and PPV of the combination of Surprise Question and ECOG score for dying within one year show similar results to Verhoef [16], who compared survival of advanced cancer patients visiting the emergency department in a Dutch academic medical center. Combing Surprise Question and ECOG Score 3–4 showed nearly the same sensitivity (40.1%), specificity (92.3%), and PPV (95.1%), but lower NPV (29.4%) for dying within one year [16]. The higher NPV in our study could be the result of collective with more patients with a higher life-expectancy: the median overall survival in our collective was higher (more than 4 months compared to 3 months). Similar to our findings, Verhoef et al. recommends triggering palliative care, if the Surprise Question is answered with “no” and ECOG score is 3–4 points [16].

One strength of our study is the use of pre-specified clinical criteria as prediction tools, rather than developing a new empirical model based on our dataset. This approach reflects routine clinical practice, where decisions are often guided by established, rule-based frameworks rather than complex statistical models. Pre-specified tools offer the advantages of transparency, interpretability, and reproducibility, and can be more easily implemented in everyday clinical workflows.

In contrast, empirically derived models—while potentially offering higher predictive accuracy in a given dataset—carry the risk of overfitting and often require validation in independent cohorts before clinical adoption.

### Limitations

There are limitations to this study. Due to the design of the previous study, we used as a database, screening score, ECOG score and IPOS score were only available for screened patients (*n* = 206) but not for all included patients (*n* = 316). Also, the previous study more than one third of the patients needed to be excluded because the questionnaire was incomplete or non-responded [5]. Besides, the diagnoses Benthien and Gaertner used to give their recommendations are not identical, thus patient cases are not congruent. This may have had an impact on the results presented here and should be considered when interpreting the generalizability of the findings. Due to the retrospective design of the study, some patient cases had to be excluded or might have had a more precise result if a prospective design was chosen.

Also, there is no gold standard tool to define SPC need neither by proxies nor by self-assessment. The standards we chose as indicators (survival, IPOS Score, preexisting contact to PC) are only an attempt to objectively capture the complex concept of SPC need. A SPC physician might be more precise in defining which patient has SPC need.

Further research is needed to define and improve more and better instruments to identify patients in need of SPC.

## Conclusions

In summary, the combination of the Surprise Question and the German NCCN tool, showed the best results identifying patients with SPC needs but is also more time-consuming than other approaches. We suggest all patients suffering from an incurable and advanced cancer should at least be screened using the Surprise Question for SPC needs. If screened positive and the ECOG score measures 3 or 4 points, SPC should be provided to the patient. Although this approach is not the most sensitive one, it is highly specific and time-efficient in identifying patients with SPC needs.

## Supplementary Information

Supplementary Material 1. Table 6: Included diagnoses according to Gaertner. Table 7: Excluded diagnoses according to Gaertner. Table 8: Included diagnoses according to Benthien. Table 9: Excluded diagnoses according to Benthien.

Supplementary Material 2. Table 10: Contingency table NCCN screening and IPOS item. Table 11: Contingency table NCCN screening and previos contact specialized palliative care. Table 12: Contingency table NCCN screening and dying within 30 days. Table 13: Contingency table NCCN screening and dying within one year. Table 14: Contingency table NCCN screening and IPOS item, previous contact with PM and dying within 30 days. Table 15: Contingency table NCCN screening and IPOS, previous contact with PM and dying within one year.

Supplementary Material 3. Figure 2: Kaplan-Meier survival curve stratified by NCCN screening score. The NCCN screening tool was applied only to patients with advanced, incurable cancer and a “No” answer to the Surprise Question. Patients with a total score of ≥5 were classified as having a need for specialized palliative care (SPC); patients with scores ≤4 were classified as having no SPC need. Survival time in days after screening is shown; censored observations are indicated accordingly. Figure 3: Kaplan-Meier survival curve stratified by palliative care need according to the Benthien criteria. The Benthien approach, developed for the DOMUS study, classifies patients as having a palliative care need based on the presence of limited antineoplastic treatment options. Patients meeting these criteria were classified as having an SPC need; all others as not having an SPC need. Survival time in days after screening is shown; censored observations are indicated accordingly. Figure 4: Kaplan-Meier survival curve stratified by the presence or absence of a need for specialized palliative care according to the Gaertner criteria.Gaertner et al. developed disease-specific guidelines for the early integration of palliative care based on tumor type, disease stage, and remaining treatment options. Patients fulfilling the Gaertner criteria were classified as having an SPC need; others were classified as not having an SPC need. Survival time in days after screening is shown; censored observations are indicated accordingly. Figure 5: Kaplan-Meier survival curve stratified by the response to the Surprise QuestionSurvival time in days after screening is shown for two groups: patients for whom the Surprise Question was answered with "No" (i.e., death would not be a surprise) and those for whom it was answered with "Yes". Censored observations are indicated accordingly. Figure 6: Kaplan-Meier survival curve stratified by ECOG performance status (0–2 vs. 3–4). Survival time in days after screening is shown for patients with ECOG scores of 0–2 and those with scores of 3–4. Censored observations are indicated accordingly. Figure 7: Kaplan-Meier survival curve stratified by combined assessment of the Surprise Question and the German NCCN screening tool.Patients were classified as having an SPC need if the Surprise Question was answered with “No” and the NCCN screening score was 5 or higher. Patients with a “Yes” answer to the Surprise Question or an NCCN score of 0–4 were classified as having no SPC need. Survival time in days after screening is shown; censored observations are indicated accordingly. Figure 8: Kaplan-Meier survival curve stratified by the combined assessment of the Surprise Question and ECOG performance status.Patients were classified as having an SPC need if the Surprise Question was answered with “No” and the ECOG score was 3–4. All others were categorized as “No SPC need”. Survival time in days after screening is shown; censored observations are indicated accordingly.

## Data Availability

All data is presented in the manuscript. The datasets analyzed during this study are available from the corresponding author on reasonable request.

## Abbreviations

ECOG: Eastern Cooperative Oncology Group
IPOS: Integrated Palliative care Outcome Scale
NCCN: National Comprehensive Cancer Network
NPV: Negative predictive value
PC: Palliative care
PPV: Positive predictive value
PROM: Patient reported outcomes
SPC: Specialized palliative care
SPSS: Statistical Package for the Social Sciences
