# Supplementary material for: Comparison of different proxy approaches to determine the need for specialized palliative care in patients with incurable cancer
Source: BMC Palliat Care. 2026 May 6;25:129. doi: 10.1186/s12904-026-02106-z (PMC13151274; doi:10.1186/s12904-026-02106-z)
Supplement: Supplementary file 1 — Supplementary Material 1. Table 6: Included diagnoses according to Gaertner. Table 7: Excluded diagnoses according to Gaertner. Table 8: Included diagnoses according to Benthien. Table 9: Excluded diagnoses according to Benthien. [file 12904_2026_2106_MOESM1_ESM.docx]

## Included Diagnoses according to Gaertner

| **Patient’s diagnosis** | **Number of cases** | **Category according to Gaertner** |
| --- | --- | --- |
| Acute myelogenous leukemia | 10 | AML |
| Astrocytoma (anaplastic) | 1 | Brain tumors |
| Breast cancer | 28 | Breast cancer |
| Bronchial carcinoma | 35 | Lung cancer |
| Central nervous system lymphoma | 1 | Brain tumors |
| Cervical cancer | 11 | Cervical cancer |
| Chronic lymphatic leukemia | 1 | CLL |
| Colon cancer | 5 | Colorectal cancer |
| Ewing’s sarcoma | 1 | Sarcoma |
| Gastric cancer | 6 | Gastric cancer |
| Glioblastoma | 3 | Brain tumors |
| Leiomyosarcoma | 2 | Sarcoma |
| Melanoma | 6 | Melanoma |
| Meningioma (anaplastic) | 1 | Brain tumors |
| Oropharyngeal cancer | 1 | Oropharyngeal cancer |
| Ovarian cancer | 8 | Ovarian cancer |
| Pancreas cancer | 10 | Pancreas cancer |
| Prostate cancer | 2 | Prostate cancer |
| Rectal cancer | 3 | Colorectal cancer |
| Tongue cancer | 1 | Oral cancer |
| Uterine cancer (sarcoma) | 3 | Sarcoma |
| Total | 139 |  |

Table 6: Included diagnoses according to Gaertner

## Excluded diagnoses according to Gaertner

| **Patient’s diagnosis** | **Number of cases** | **Reason for exclusion** |
| --- | --- | --- |
| Cancer of unknown primary origin | 2 | No recommendation for this diagnosis available |
| Carcinoma of the bladder | 7 | No recommendation for this diagnosis available |
| Cholangiocarcinoma | 2 | No recommendation for this diagnosis available |
| Esophageal cancer | 8 | No recommendation for this diagnosis available |
| Esophagogastric junction cancer | 1 | No recommendation for this diagnosis available |
| Fallopian tube carcinoma | 1 | No recommendation for this diagnosis available |
| Floor of mouth cancer | 1 | Progress likely but not documented |
| Gastric and colon cancer | 1 | No recommendation for this diagnosis available |
| Hepatocellular carcinoma | 4 | No recommendation for this diagnosis available |
| Hypopharyngeal carcinoma | 1 | No recommendation for this diagnosis available |
| Laryngeal carcinoma | 2 | No recommendation for this diagnosis available |
| Lymphoma | 8 | No recommendation for this diagnosis available |
| Merkel-cell carcinoma | 1 | No recommendation for this diagnosis available |
| Multiple myeloma | 10 | No recommendation for this diagnosis available |
| Orbital cancer | 1 | No recommendation for this diagnosis available |
| Oropharyngeal cancer | 1 | Missing information in patient record |
| Pleural mesothelioma | 1 | No recommendation for this diagnosis available |
| Renal cell carcinoma | 11 | No recommendation for this diagnosis available |
| Squamous cell skin cancer | 1 | No recommendation for this diagnosis available |
| Thyme cancer | 1 | No recommendation for this diagnosis available |
| Uterine cancer (adenocarcinoma) | 1 | No recommendation for this diagnosis available |
| Uterine cancer (endometrial carcinoma) | 1 | No recommendation for this diagnosis available |
| Total | 67 |  |

Table 7: Excluded diagnoses according to Gaertner

## Included diagnoses according to Benthien

| **Patient’s diagnosis** | **Number of cases** | **Category according to Benthien** |
| --- | --- | --- |
| Astrocytoma (anaplastic) | 1 | CNS cancer |
| Breast cancer | 26 | Breast cancer |
| Bronchial carcinoma | 33 | Lung cancer |
| Cholangiocarcinoma | 2 | Gastrointestinal cancer |
| Colon cancer | 5 | Gastrointestinal cancer |
| Cancer of unknown primary origin CUP | 1 | Cancer of unknown primary origin |
| Esophagogastric junction cancer | 1 | Gastrointestinal cancer |
| Glioblastoma | 3 | CNS cancer |
| Carcinoma of the bladder | 6 | Prostate, penile, bladder or thyme cancer or adrenal carcinoma |
| Hepatocellular carcinoma | 3 | Gastrointestinal cancer |
| Hypopharyngeal carcinoma | 1 | Head and neck cancer |
| Laryngeal carcinoma | 2 | Head and neck cancer |
| Gastric cancer | 6 | Gastrointestinal cancer |
| Gastric and colon cancer | 1 | Gastrointestinal cancer |
| Meningioma (anaplastic) | 1 | CNS cancer |
| Esophageal cancer | 8 | Gastrointestinal cancer |
| Oropharyngeal cancer | 1 | Head and neck cancer |
| Ovarian cancer | 8 | Ovarian or uterine cancer |
| Pancreas cancer | 9 | Gastrointestinal cancer |
| Prostate cancer | 2 | Prostate, penile, bladder or thyme cancer or adrenal carcinoma |
| Rectal cancer | 3 | Gastrointestinal cancer |
| Thyme cancer | 1 | Prostate, penile, bladder or thyme cancer or adrenal carcinoma |
| Uterine cancer (adenocarcinoma) | 1 | Ovarian or uterine cancer |
| Uterine cancer (endometrial carcinoma) | 1 | Ovarian or uterine cancer |
| Uterine cancer (sarcoma) | 3 | Ovarian or uterine cancer |
| Cervical cancer | 11 | Cervical or vulva cancer |
| Central nervous system lymphoma | 1 | CNS cancer |
| Tongue cancer | 1 | Head and neck cancer |
| Total | 142 |  |

Table 8: Included diagnoses according to Benthien

## Excluded diagnoses according to Benthien

| **Patient’s diagnosis** | **Number of cases** | **Reason for exclusion** |
| --- | --- | --- |
| Breast cancer | 2 | Progress likely but not documented |
| Bronchial carcinoma | 2 | Progress likely but not documented |
| Cancer of unknown primary origin CUP | 1 | Missing information in patient record |
| Carcinoma of the bladder | 1 | Missing information in patient record |
| Ewing’s sarcoma | 1 | No recommendation for this diagnosis available |
| Fallopian tube carcinoma | 1 | No recommendation for this diagnosis available |
| Floor of mouth cancer | 1 | Progress likely but not documented |
| Hepatocellular carcinoma | 1 | Progress likely but not documented |
| Leiomyosarcoma | 2 | No recommendation for this diagnosis available |
| Leukemia | 11 | No recommendation for this diagnosis available |
| Lymphoma | 8 | No recommendation for this diagnosis available |
| Melanoma | 6 | No recommendation for this diagnosis available |
| Merkel-cell carcinoma | 1 | No recommendation for this diagnosis available |
| Multiple myeloma | 10 | No recommendation for this diagnosis available |
| Orbital cancer | 1 | No recommendation for this diagnosis available |
| Oropharyngeal cancer | 1 | Missing information in patient record |
| Pancreas cancer | 1 | Progress likely but not documented |
| Pleural mesothelioma | 1 | No recommendation for this diagnosis available |
| Renal cell carcinoma | 11 | No recommendation for this diagnosis available |
| Squamous cell skin cancer | 1 | No recommendation for this diagnosis available |
| Total | 64 |  |

Table 9: Excluded diagnoses according to Benthien
