# Supplementary material for: Comparison of different proxy approaches to determine the need for specialized palliative care in patients with incurable cancer
Source: BMC Palliat Care. 2026 May 6;25:129. doi: 10.1186/s12904-026-02106-z (PMC13151274; doi:10.1186/s12904-026-02106-z)
Supplement: Supplementary file 2 — Supplementary Material 2. Table 10: Contingency table NCCN screening and IPOS item. Table 11: Contingency table NCCN screening and previos contact specialized palliative care. Table 12: Contingency table NCCN screening and dying within 30 days. Table 13: Contingency table NCCN screening and dying within one year. Table 14: Contingency table NCCN screening and IPOS item, previous contact with PM and dying within 30 days. Table 15: Contingency table NCCN screening and IPOS, previous contact with PM and dying within one year. [file 12904_2026_2106_MOESM2_ESM.docx]

## Contingency tables NCCN screening

The following is an illustrative 2x2 contingency table used to calculate the sensitivity, specificity, PPV, and NPV of the NCCN screening tool.

|  | Does the calculated NCCN screening score indicate a need for palliative care (cut-off = 5 points)? | |
| --- | --- | --- |
| Did the patient score 3 or 4 points on one or more IPOS items? | Yes | No |
| Yes | 154 | 23 |
| No | 12 | 17 |

Table 10: Contingency table NCCN screening and IPOS item

|  | Does the calculated NCCN screening score indicate a need for palliative care (cut-off = 5 points)? | |
| --- | --- | --- |
| Did the patient have any previous contact with specialized palliative care? | Yes | No |
| Yes | 66 | 0 |
| No | 96 | 39 |

Table 11: Contingency table NCCN screening and previos contact specialized palliative care

|  | Does the calculated NCCN screening score indicate a need for palliative care (cut-off = 5 points)? | |
| --- | --- | --- |
| Did the patient die within 30 days? | Yes | No |
| Yes | 72 | 3 |
| No | 94 | 37 |

Table 12: Contingency table NCCN screening and dying within 30 days

|  | Does the calculated NCCN screening score indicate a need for palliative care (cut-off = 5 points)? | |
| --- | --- | --- |
| Did the patient die within one year? | Yes | No |
| Yes | 136 | 25 |
| No | 28 | 15 |

Table 13: Contingency table NCCN screening and dying within one year

|  | Does the calculated NCCN screening score indicate a need for palliative care (cut-off = 5 points)? | |
| --- | --- | --- |
| Did the patient meet any of the following criteria: scored 3 or 4 on at least one IPOS item, had previous contact with specialized palliative care, or died within 30 days? | Yes | No |
| Yes | 155 | 24 |
| No | 11 | 16 |

Table 14: Contingency table NCCN screening and IPOS item, previous contact with PM and dying within 30 days

|  | Does the calculated NCCN screening score indicate a need for palliative care (cut-off = 5 points)? | |
| --- | --- | --- |
| Did the patient meet any of the following criteria: scored 3 or 4 on at least one IPOS item, had previous contact with specialized palliative care, or died within one year? | Yes | No |
| Yes | 161 | 32 |
| No | 5 | 8 |

Table 15: Contingency table NCCN screening and IPOS, previous contact with PM and dying within one year
