# Supplementary figures and images for: Comparison of different proxy approaches to determine the need for specialized palliative care in patients with incurable cancer
Source: BMC Palliat Care. 2026 May 6;25:129. doi: 10.1186/s12904-026-02106-z (PMC13151274; doi:10.1186/s12904-026-02106-z)

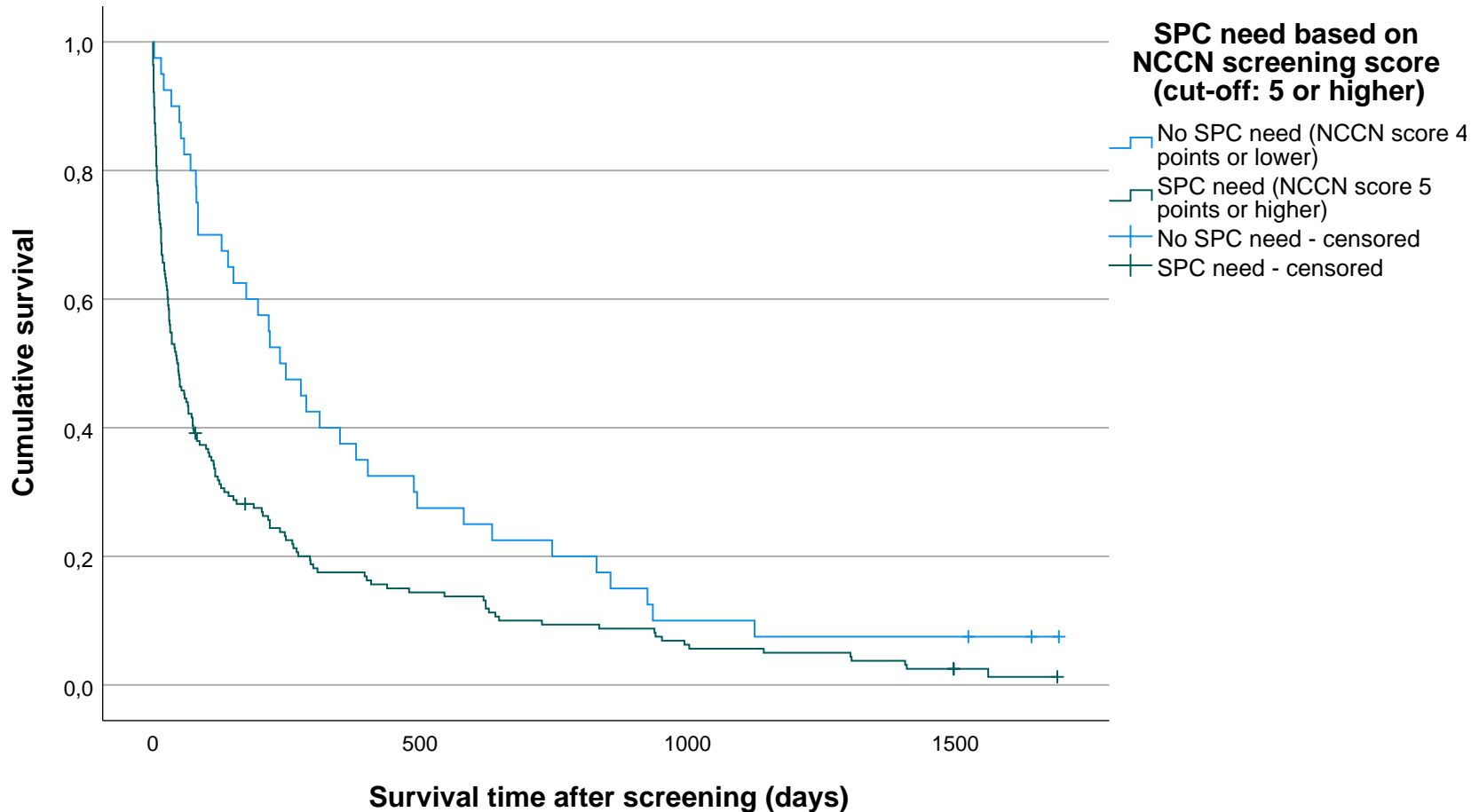

Supplement: Supplementary file 3 — Supplementary Material 3. Figure 2: Kaplan-Meier survival curve stratified by NCCN screening score. The NCCN screening tool was applied only to patients with advanced, incurable cancer and a “No” answer to the Surprise Question. Patients with a total score of ≥5 were classified as having a need for specialized palliative care (SPC); patients with scores ≤4 were classified as having no SPC need. Survival time in days after screening is shown; censored observations are indicated accordingly. Figure 3: Kaplan-Meier survival curve stratified by palliative care need according to the Benthien criteria. The Benthien approach, developed for the DOMUS study, classifies patients as having a palliative care need based on the presence of limited antineoplastic treatment options. Patients meeting these criteria were classified as having an SPC need; all others as not having an SPC need. Survival time in days after screening is shown; censored observations are indicated accordingly. Figure 4: Kaplan-Meier survival curve stratified by the presence or absence of a need for specialized palliative care according to the Gaertner criteria.Gaertner et al. developed disease-specific guidelines for the early integration of palliative care based on tumor type, disease stage, and remaining treatment options. Patients fulfilling the Gaertner criteria were classified as having an SPC need; others were classified as not having an SPC need. Survival time in days after screening is shown; censored observations are indicated accordingly. Figure 5: Kaplan-Meier survival curve stratified by the response to the Surprise QuestionSurvival time in days after screening is shown for two groups: patients for whom the Surprise Question was answered with "No" (i.e., death would not be a surprise) and those for whom it was answered with "Yes". Censored observations are indicated accordingly. Figure 6: Kaplan-Meier survival curve stratified by ECOG performance status (0–2 vs. 3–4). Survival time in days after [file 12904_2026_2106_MOESM3_ESM.zip › Figure 2.pdf]

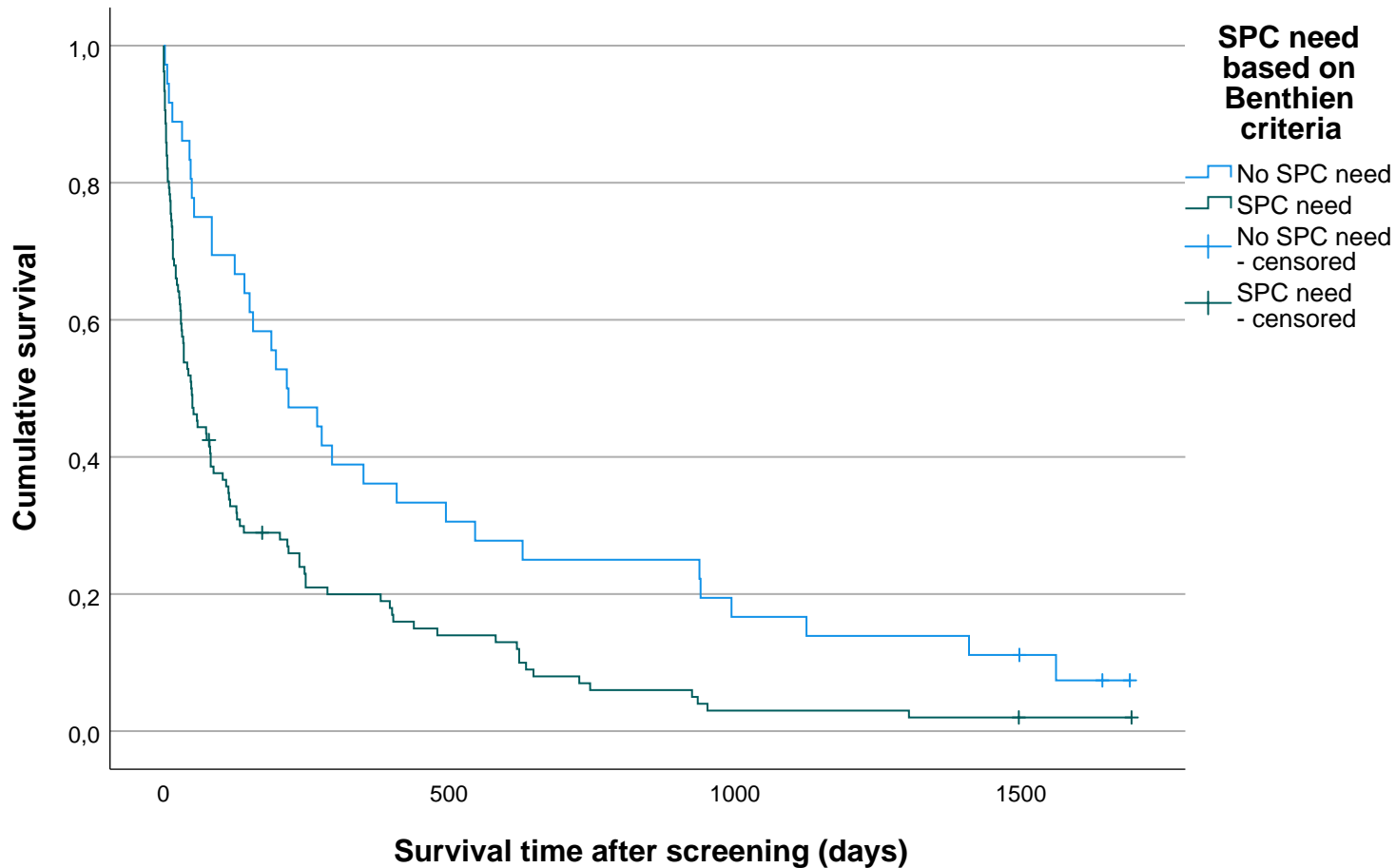

Supplement: Supplementary file 3 — Supplementary Material 3. Figure 2: Kaplan-Meier survival curve stratified by NCCN screening score. The NCCN screening tool was applied only to patients with advanced, incurable cancer and a “No” answer to the Surprise Question. Patients with a total score of ≥5 were classified as having a need for specialized palliative care (SPC); patients with scores ≤4 were classified as having no SPC need. Survival time in days after screening is shown; censored observations are indicated accordingly. Figure 3: Kaplan-Meier survival curve stratified by palliative care need according to the Benthien criteria. The Benthien approach, developed for the DOMUS study, classifies patients as having a palliative care need based on the presence of limited antineoplastic treatment options. Patients meeting these criteria were classified as having an SPC need; all others as not having an SPC need. Survival time in days after screening is shown; censored observations are indicated accordingly. Figure 4: Kaplan-Meier survival curve stratified by the presence or absence of a need for specialized palliative care according to the Gaertner criteria.Gaertner et al. developed disease-specific guidelines for the early integration of palliative care based on tumor type, disease stage, and remaining treatment options. Patients fulfilling the Gaertner criteria were classified as having an SPC need; others were classified as not having an SPC need. Survival time in days after screening is shown; censored observations are indicated accordingly. Figure 5: Kaplan-Meier survival curve stratified by the response to the Surprise QuestionSurvival time in days after screening is shown for two groups: patients for whom the Surprise Question was answered with "No" (i.e., death would not be a surprise) and those for whom it was answered with "Yes". Censored observations are indicated accordingly. Figure 6: Kaplan-Meier survival curve stratified by ECOG performance status (0–2 vs. 3–4). Survival time in days after [file 12904_2026_2106_MOESM3_ESM.zip › Figure 3.pdf]

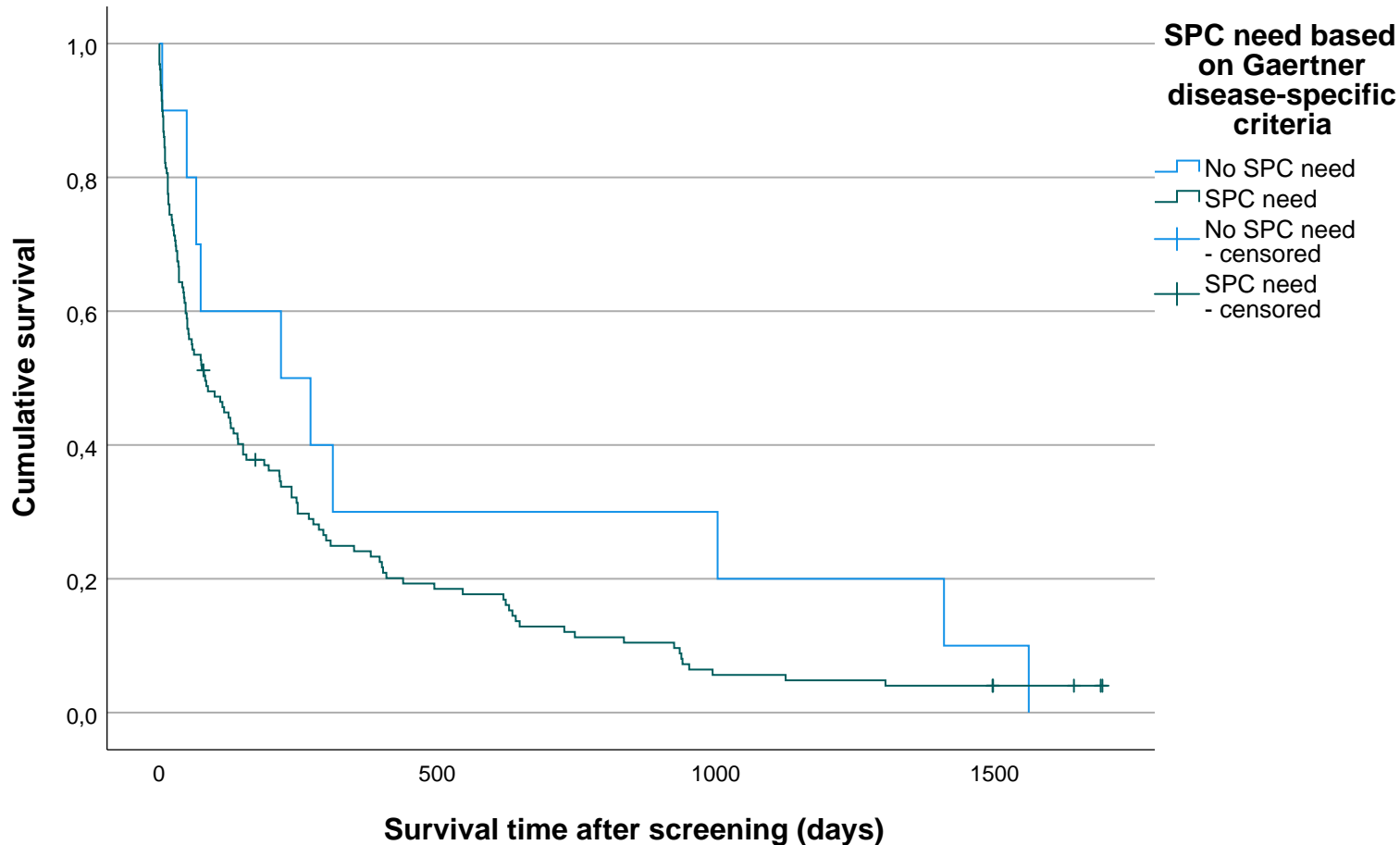

Supplement: Supplementary file 3 — Supplementary Material 3. Figure 2: Kaplan-Meier survival curve stratified by NCCN screening score. The NCCN screening tool was applied only to patients with advanced, incurable cancer and a “No” answer to the Surprise Question. Patients with a total score of ≥5 were classified as having a need for specialized palliative care (SPC); patients with scores ≤4 were classified as having no SPC need. Survival time in days after screening is shown; censored observations are indicated accordingly. Figure 3: Kaplan-Meier survival curve stratified by palliative care need according to the Benthien criteria. The Benthien approach, developed for the DOMUS study, classifies patients as having a palliative care need based on the presence of limited antineoplastic treatment options. Patients meeting these criteria were classified as having an SPC need; all others as not having an SPC need. Survival time in days after screening is shown; censored observations are indicated accordingly. Figure 4: Kaplan-Meier survival curve stratified by the presence or absence of a need for specialized palliative care according to the Gaertner criteria.Gaertner et al. developed disease-specific guidelines for the early integration of palliative care based on tumor type, disease stage, and remaining treatment options. Patients fulfilling the Gaertner criteria were classified as having an SPC need; others were classified as not having an SPC need. Survival time in days after screening is shown; censored observations are indicated accordingly. Figure 5: Kaplan-Meier survival curve stratified by the response to the Surprise QuestionSurvival time in days after screening is shown for two groups: patients for whom the Surprise Question was answered with "No" (i.e., death would not be a surprise) and those for whom it was answered with "Yes". Censored observations are indicated accordingly. Figure 6: Kaplan-Meier survival curve stratified by ECOG performance status (0–2 vs. 3–4). Survival time in days after [file 12904_2026_2106_MOESM3_ESM.zip › Figure 4.pdf]

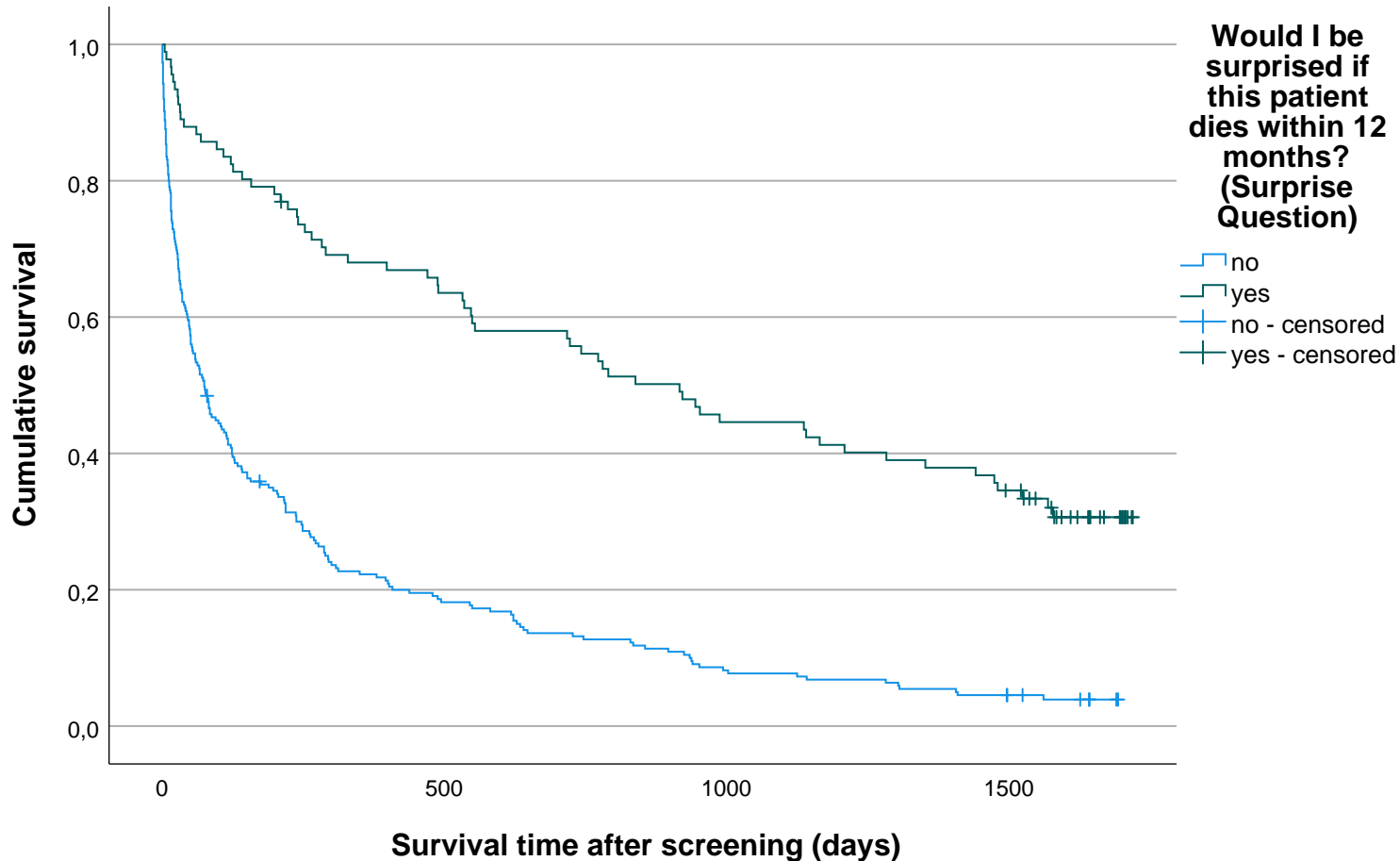

Supplement: Supplementary file 3 — Supplementary Material 3. Figure 2: Kaplan-Meier survival curve stratified by NCCN screening score. The NCCN screening tool was applied only to patients with advanced, incurable cancer and a “No” answer to the Surprise Question. Patients with a total score of ≥5 were classified as having a need for specialized palliative care (SPC); patients with scores ≤4 were classified as having no SPC need. Survival time in days after screening is shown; censored observations are indicated accordingly. Figure 3: Kaplan-Meier survival curve stratified by palliative care need according to the Benthien criteria. The Benthien approach, developed for the DOMUS study, classifies patients as having a palliative care need based on the presence of limited antineoplastic treatment options. Patients meeting these criteria were classified as having an SPC need; all others as not having an SPC need. Survival time in days after screening is shown; censored observations are indicated accordingly. Figure 4: Kaplan-Meier survival curve stratified by the presence or absence of a need for specialized palliative care according to the Gaertner criteria.Gaertner et al. developed disease-specific guidelines for the early integration of palliative care based on tumor type, disease stage, and remaining treatment options. Patients fulfilling the Gaertner criteria were classified as having an SPC need; others were classified as not having an SPC need. Survival time in days after screening is shown; censored observations are indicated accordingly. Figure 5: Kaplan-Meier survival curve stratified by the response to the Surprise QuestionSurvival time in days after screening is shown for two groups: patients for whom the Surprise Question was answered with "No" (i.e., death would not be a surprise) and those for whom it was answered with "Yes". Censored observations are indicated accordingly. Figure 6: Kaplan-Meier survival curve stratified by ECOG performance status (0–2 vs. 3–4). Survival time in days after [file 12904_2026_2106_MOESM3_ESM.zip › Figure 5.pdf]

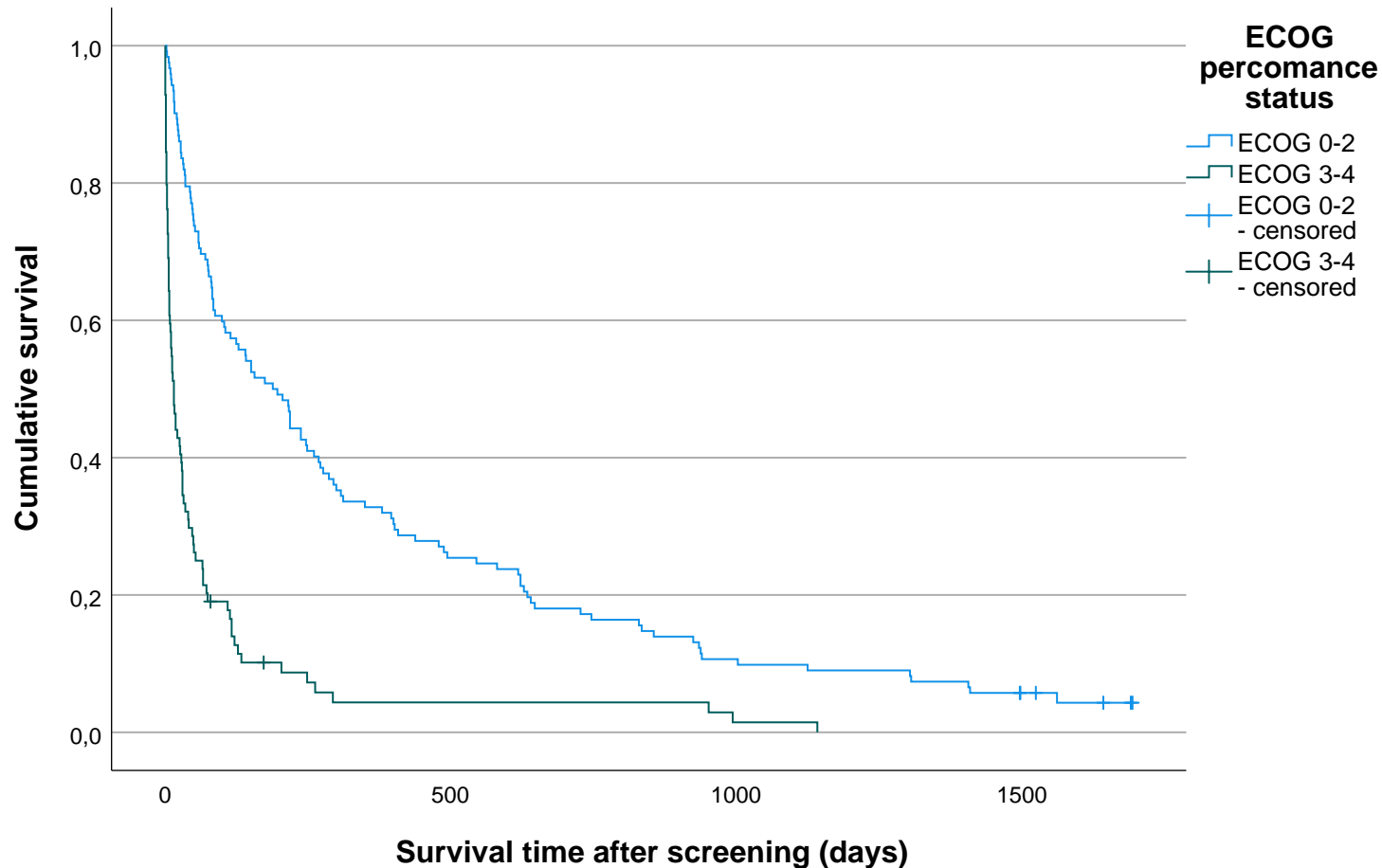

Supplement: Supplementary file 3 — Supplementary Material 3. Figure 2: Kaplan-Meier survival curve stratified by NCCN screening score. The NCCN screening tool was applied only to patients with advanced, incurable cancer and a “No” answer to the Surprise Question. Patients with a total score of ≥5 were classified as having a need for specialized palliative care (SPC); patients with scores ≤4 were classified as having no SPC need. Survival time in days after screening is shown; censored observations are indicated accordingly. Figure 3: Kaplan-Meier survival curve stratified by palliative care need according to the Benthien criteria. The Benthien approach, developed for the DOMUS study, classifies patients as having a palliative care need based on the presence of limited antineoplastic treatment options. Patients meeting these criteria were classified as having an SPC need; all others as not having an SPC need. Survival time in days after screening is shown; censored observations are indicated accordingly. Figure 4: Kaplan-Meier survival curve stratified by the presence or absence of a need for specialized palliative care according to the Gaertner criteria.Gaertner et al. developed disease-specific guidelines for the early integration of palliative care based on tumor type, disease stage, and remaining treatment options. Patients fulfilling the Gaertner criteria were classified as having an SPC need; others were classified as not having an SPC need. Survival time in days after screening is shown; censored observations are indicated accordingly. Figure 5: Kaplan-Meier survival curve stratified by the response to the Surprise QuestionSurvival time in days after screening is shown for two groups: patients for whom the Surprise Question was answered with "No" (i.e., death would not be a surprise) and those for whom it was answered with "Yes". Censored observations are indicated accordingly. Figure 6: Kaplan-Meier survival curve stratified by ECOG performance status (0–2 vs. 3–4). Survival time in days after [file 12904_2026_2106_MOESM3_ESM.zip › Figure 6.pdf]

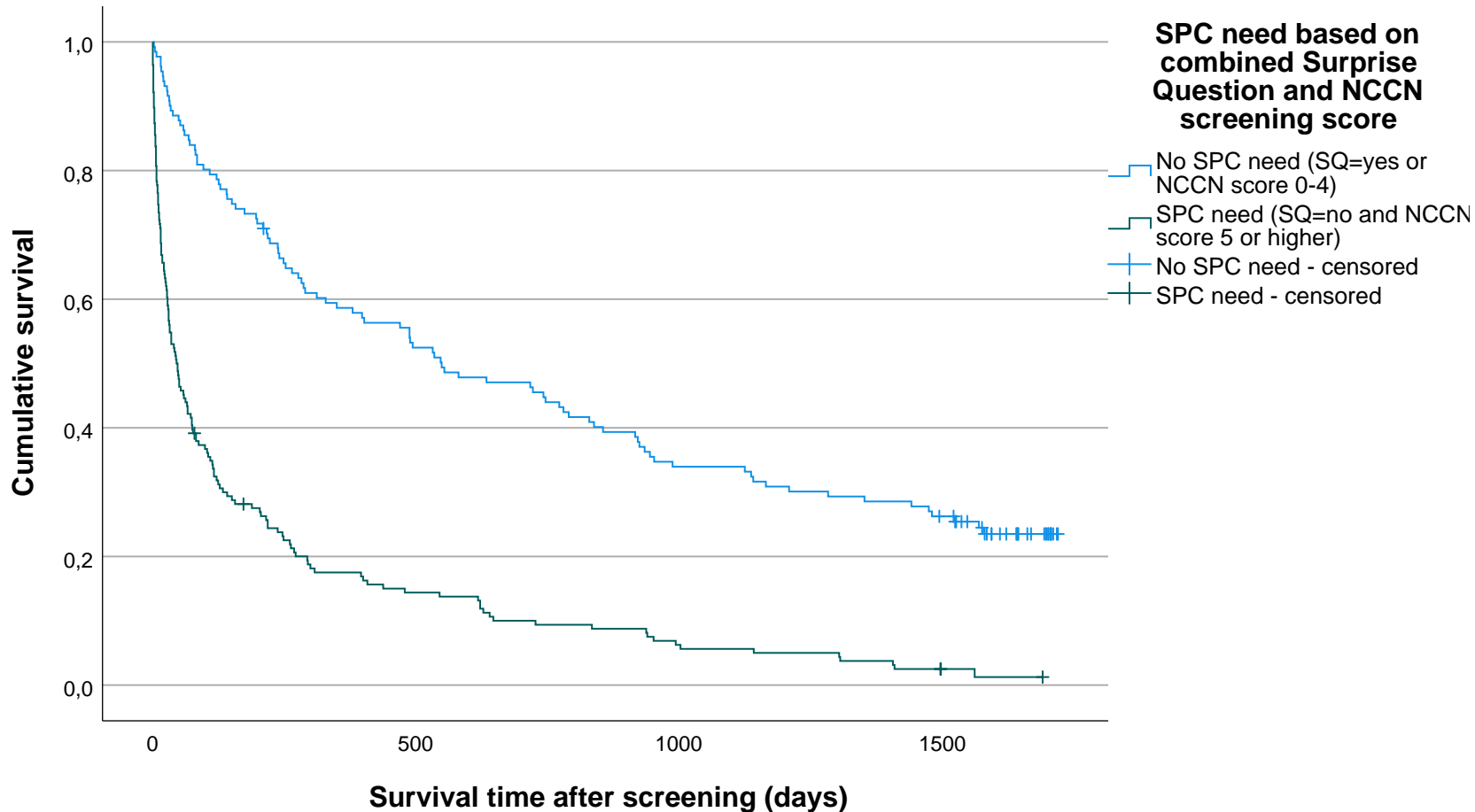

Supplement: Supplementary file 3 — Supplementary Material 3. Figure 2: Kaplan-Meier survival curve stratified by NCCN screening score. The NCCN screening tool was applied only to patients with advanced, incurable cancer and a “No” answer to the Surprise Question. Patients with a total score of ≥5 were classified as having a need for specialized palliative care (SPC); patients with scores ≤4 were classified as having no SPC need. Survival time in days after screening is shown; censored observations are indicated accordingly. Figure 3: Kaplan-Meier survival curve stratified by palliative care need according to the Benthien criteria. The Benthien approach, developed for the DOMUS study, classifies patients as having a palliative care need based on the presence of limited antineoplastic treatment options. Patients meeting these criteria were classified as having an SPC need; all others as not having an SPC need. Survival time in days after screening is shown; censored observations are indicated accordingly. Figure 4: Kaplan-Meier survival curve stratified by the presence or absence of a need for specialized palliative care according to the Gaertner criteria.Gaertner et al. developed disease-specific guidelines for the early integration of palliative care based on tumor type, disease stage, and remaining treatment options. Patients fulfilling the Gaertner criteria were classified as having an SPC need; others were classified as not having an SPC need. Survival time in days after screening is shown; censored observations are indicated accordingly. Figure 5: Kaplan-Meier survival curve stratified by the response to the Surprise QuestionSurvival time in days after screening is shown for two groups: patients for whom the Surprise Question was answered with "No" (i.e., death would not be a surprise) and those for whom it was answered with "Yes". Censored observations are indicated accordingly. Figure 6: Kaplan-Meier survival curve stratified by ECOG performance status (0–2 vs. 3–4). Survival time in days after [file 12904_2026_2106_MOESM3_ESM.zip › Figure 7.pdf]

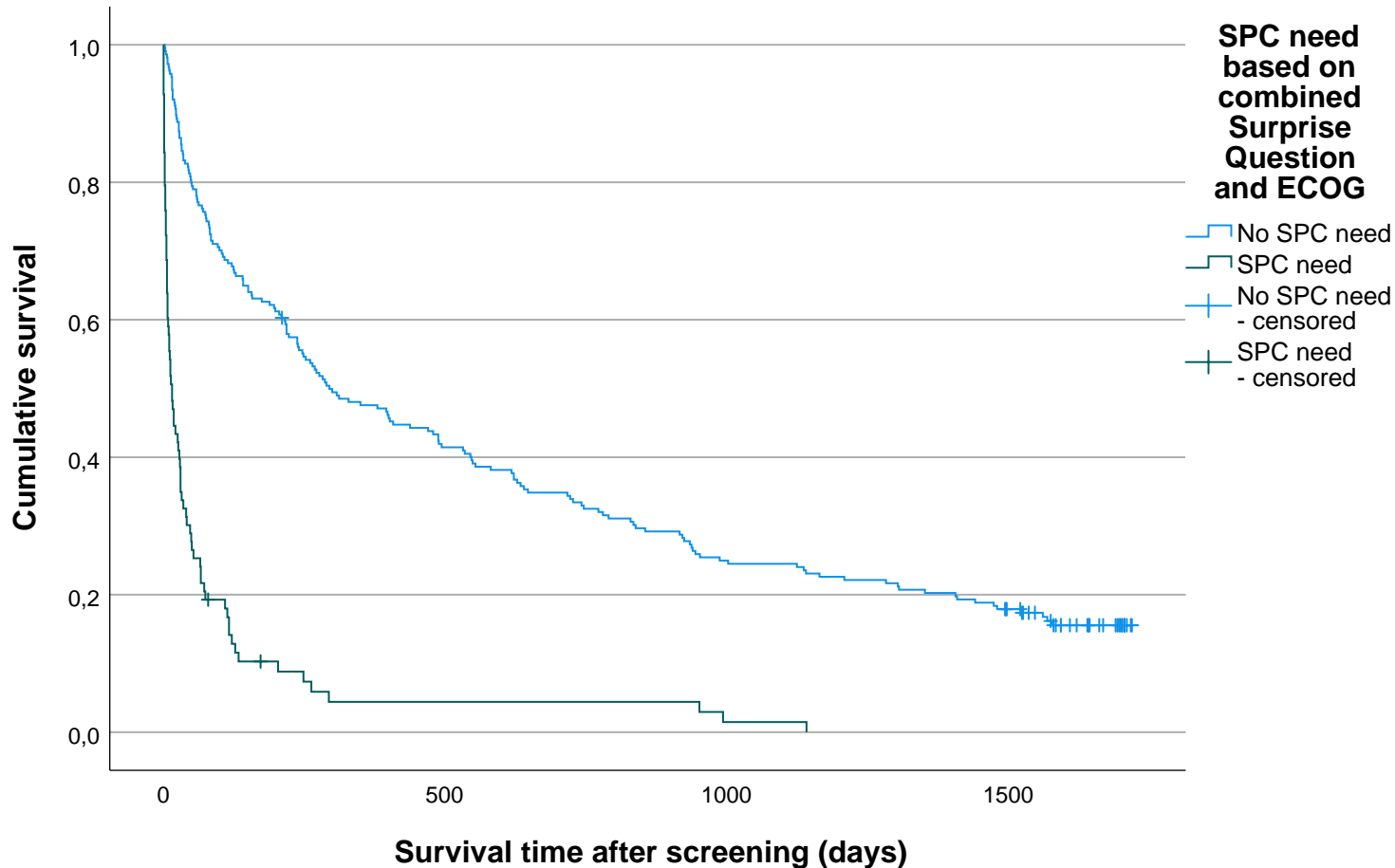

Supplement: Supplementary file 3 — Supplementary Material 3. Figure 2: Kaplan-Meier survival curve stratified by NCCN screening score. The NCCN screening tool was applied only to patients with advanced, incurable cancer and a “No” answer to the Surprise Question. Patients with a total score of ≥5 were classified as having a need for specialized palliative care (SPC); patients with scores ≤4 were classified as having no SPC need. Survival time in days after screening is shown; censored observations are indicated accordingly. Figure 3: Kaplan-Meier survival curve stratified by palliative care need according to the Benthien criteria. The Benthien approach, developed for the DOMUS study, classifies patients as having a palliative care need based on the presence of limited antineoplastic treatment options. Patients meeting these criteria were classified as having an SPC need; all others as not having an SPC need. Survival time in days after screening is shown; censored observations are indicated accordingly. Figure 4: Kaplan-Meier survival curve stratified by the presence or absence of a need for specialized palliative care according to the Gaertner criteria.Gaertner et al. developed disease-specific guidelines for the early integration of palliative care based on tumor type, disease stage, and remaining treatment options. Patients fulfilling the Gaertner criteria were classified as having an SPC need; others were classified as not having an SPC need. Survival time in days after screening is shown; censored observations are indicated accordingly. Figure 5: Kaplan-Meier survival curve stratified by the response to the Surprise QuestionSurvival time in days after screening is shown for two groups: patients for whom the Surprise Question was answered with "No" (i.e., death would not be a surprise) and those for whom it was answered with "Yes". Censored observations are indicated accordingly. Figure 6: Kaplan-Meier survival curve stratified by ECOG performance status (0–2 vs. 3–4). Survival time in days after [file 12904_2026_2106_MOESM3_ESM.zip › Figure 8.pdf]
